# The Beijing version of the montreal cognitive assessment as a brief screening tool for mild cognitive impairment: a community-based study

**DOI:** 10.1186/1471-244X-12-156

**Published:** 2012-09-25

**Authors:** Jing Yu, Juan Li, Xin Huang

**Affiliations:** 1 Center on Ageing Psychology, Key Laboratory of Mental Health, Institute of Psychology, Chinese Academy of Sciences, 4A Datun Road, Chaoyang District, Beijing, 100101, China; 2 Graduate School, Chinese Academy of Sciences, Beijing, China; 3 Faculty of Psychology, Southwest University, Chongqing, China

**Keywords:** MoCA-BJ, MMSE, Mild cognitive impairment, Dementia, Cognitive assessment

## Abstract

**Background:**

A cross-sectional validation study was conducted in several urban and rural communities in Beijing, China, to evaluate the effectiveness of the Beijing version of the Montreal Cognitive Assessment (MoCA-BJ) as a screening tool to detect mild cognitive impairment (MCI) among Chinese older adults.

**Methods:**

The MoCA-BJ and the Mini-Mental State Examination (MMSE) were administered to 1001 Chinese elderly community dwellers recruited from three different regions (i.e., newly developed, old down-town, and rural areas) in Beijing. Twenty-one of these participants were diagnosed by experienced psychiatrists as having dementia, 115 participants were diagnosed as MCI, and 865 participants were considered to be cognitively normal. To analyze the effectiveness of the MoCA-BJ, we examined its psychometric properties, conducted item analyses, evaluated the sensitivity and specificity of the scale, and compared the scale with the MMSE. Demographic and regional differences among our subjects were also taken into consideration.

**Results:**

Under the recommended cut-off score of 26, the MoCA-BJ demonstrated an excellent sensitivity of 90.4%, and a fair specificity (31.3%). The MoCA-BJ showed optimal sensitivity (68.7%) and specificity (63.9%) when the cut-off score was lowered to 22. Among all the seven cognitive sub-domains, delayed recall was shown to be the best index to differentiate MCI from the normal controls. Regional differences disappeared when the confounding demographic variables (i.e., age and education) were controlled. Item analysis showed that the internal consistency was relatively low in both naming and sentence repetition tasks, and the diagnostic accuracy was similar between the MoCA-BJ and the MMSE.

**Conclusions:**

In general, the MoCA-BJ is an acceptable tool for MCI screening in both urban and rural regions of Beijing. However, presumably due to the linguistic and cultural differences between the original English version and the Chinese version of the scale, and the lower education level of Chinese older adults, the MoCA-BJ is not much better than the MMSE in detecting MCI, at least for this study sample. Further modifications to several test items of the MoCA-BJ are recommended in order to improve the applicability and effectiveness of the MoCA-BJ in MCI screening among the Chinese population.

## Background

Similar to many other developing countries, the aging population has been growing rapidly in China. According to the latest National Population Census [1], there are approximately 178 million older adults in China up to 2010. It is therefore not surprising that the epidemic of aging-related diseases is also rising rapidly in the country. One of these diseases is dementia, which tremendously affects the quality of life of older adults and brings upon a number of related economic and public health issues. Early detection of and effective intervention for dementia are crucial. Recently, researchers have identified a pre-dementia syndrome, mild cognitive impairment (MCI) [2], which is an intermediate clinical state between normal aging and dementia. The diagnosis of and intervention for MCI could be an effective way for early detection and decelerating the progress of dementia among older adults. In order to improve the diagnostic accuracy of MCI, the use of a combination of neuropsychological assessment batteries, biomarker tests, and neuroimaging techniques was proposed. However, given the cost and time needed, it is often not feasible to use neuroimaging techniques (e.g., MRI, PET, DTI, etc.) or comprehensive neuropsychological tests (e.g., Global Deterioration Scale, GDS [3], Clinical Dementia Rating, CDR [4], etc.) in a community setting. A brief screening tool would therefore be a more practical approach for frontline clinicians to detect MCI. The Mini-Mental State Examination (MMSE) [5] is the most widely used screening tool for dementia; however, it has been demonstrated to be less sensitive to MCI. Other than the MMSE, the Montreal Cognitive Assessment (MoCA) [6] was specifically developed as a screening tool for MCI and mild dementia, and has been shown to have high sensitivity and specificity for differentiating individuals with MCI from healthy individuals in several developed countries and areas [6-9].

There are five Chinese versions of the MoCA, including the Beijing version, the Changsha version, the Guangdong version, the Hong Kong version, and the Taiwan version (the specific test forms and instructions for each version are available at the MoCA official website http://www.mocatest.org/). Among these versions, there are some subtle variations in the employed language (i.e., Mandarin vs. Cantonese), the stimuli used in the trail making task (i.e., Arabic numbers interfered with Chinese sequence-meaning characters vs. Color trail test [10]), the semantic category used in the verbal fluency task (i.e., animals vs. vegetables), and the specific words and pictures used in the delayed recall and naming task respectively [11]. The most widely used version of MoCA in mainland China is the Beijing version (MoCA-BJ). Given that elderly in China, similar to those in many other Asian countries [8,12], have relatively low education level, and that the MoCA scale is translated based on the original Western version with some linguistic and cultural modifications, it remains uncertain whether the MoCA-BJ could be effectively applied to the Chinese community-based population. Although the reliability and validity of the MoCA-BJ were evaluated in several studies published in Chinese journals, most of them were clinically-based, were sampled from nursing homes rather than from community-based settings [13-15], or have relatively small sample sizes [16,17]. Moreover, regional differences (e.g., urban versus rural) have rarely been compared in China, despite the fact that 72.5% of elderly in mainland China live in rural areas [18]. Recently, a study investigated the use of the MoCA-BJ in five regions (Beijing, Zhengzhou, Guangzhou, Changchun, and Guiyang) of mainland China and found high sensitivity (83.8%) and specificity (82.5%) in the MoCA-BJ for detection of MCI [19]. However, to our knowledge, this is the only study examining the use of MoCA-BJ in different regions of China with a large sample size, and the MCI prevalence in this study (approximately 20.0%) is higher than the pooled MCI prevalence (12.7%) in the Chinese population as reported in a recent meta-analysis [20]. Therefore, there is still a need to validate the effectiveness of the MoCA-BJ in China with a large sample size.

The goal of the current study is to examine the sensitivity and specificity of the MoCA-BJ as a screening tool for MCI in a community-based population residing in both urban and rural areas of Beijing.

## Methods

### The Beijing version of MoCA (MoCA-BJ)

The MoCA-BJ used in current study is one of the five Chinese versions of MoCA, and has been translated and used in previous studies with clinical populations. The items that are used to examine the seven cognitive domains (i.e. visuospatial/executive function, naming, attention, abstraction, language, delayed memory, and orientation) are translated from the original English version literally, with the exception of the following modifications:

1) Visuospatial/executive function domain: The alphabet letters are replaced by Chinese characters (甲/乙/丙/丁/戊) which contain the same sequential meanings as “A/B/C/D/E” in English.

2) Attention domain: Numbers are used instead of English alphabet letters in the auditory vigilance task.

3) Language domain: In the verbal fluency task, the phonemic fluency task that requires participants to generate words beginning with the letter F is replaced by the semantic fluency task requiring participants to produce as many animals as possible in sixty seconds.

### Data collection

As residents in rural areas may have lower socioeconomic status (SES), less access to medical facilities etc., which could interfere with their performance on neuropsychological tests [19], the validation of MoCA as a diagnostic assessment may need to be conducted in urban and rural areas separately. In addition, with the rapid socioeconomic transitions taking place in Beijing, even within urban areas, the newly developed areas are also quite different from old down-town areas, as the residents in newly developed areas usually have higher SES and better living conditions etc. Therefore, ChaoYang, XiCheng, and ChangPing Districts were selected to represent the newly developed (i.e., New Town), old downtown (i.e., Old Town), and rural areas (i.e., Rural Area) of Beijing, respectively. The sample sizes for the three regions were determined by the actual distribution of older adults residing in the three regions in Beijing, which are 57.0%, 20.7%, and 22.3% for New Town, Old Town, and Rural Area, respectively (data reported by Beijing Government Council on Aging in 2011, from http://zhengwu.beijing.gov.cn/tjxx/tjgb/P020111124398948185702.pdf). Three communities from ChaoYang District, one community from XiCheng District, and two villages from Chang Ping District were then conveniently selected to recruit the participants from. Residents listed in the census of the community registration that were aged 60 and above were contacted for participation. One thousand and fifty-six participants participated in the present study, and 1001 participants were included in the final data analyses based on the following inclusion and exclusion criteria. Inclusion criteria were individuals (1) who were 60 years old or older and registered as permanent residents in their residing district in Beijing (n = 1056), and (2) who completed both the MoCA-BJ and the MMSE (n = 1036). Exclusion criteria were individuals (1) who had missing clinical diagnoses (n = 25), and (2) who had received a clinical diagnosis of depression (n = 10).

This study was approved by the ethics committees of the Institute of Psychology, Chinese Academy of Science. Informed consent was obtained from each participant or their care-givers for those who suffered from dementia and other kinds of disabilities that have prevented them from signing the consent forms themselves.

### Procedures and participants

All participants completed a battery of neuropsychological tests (including MoCA-BJ and MMSE), clinical assessment, laboratory tests, and neuroimaging examinations when applicable. The clinical assessment included a survey on participants’ medical history, a basic physical exam, as well as the Neuropsychiatric Inventory (NPI), the Activities of Daily Life (ADL), the Global Deterioration Scale (GDS), the Clinical Dementia Rating (CDR), the Hachinski Ischemic Score (HIS), and the Structured Clinical Interview for DSM Disorders (SCID, depression and anxiety parts only). Research assistants with psychological background administered the neuropsychological battery, and psychiatrists were responsible for the clinical assessment. All the research assistants and clinicians were intensively trained. High inter-rater reliability (above 90%) was obtained with the support of a consensus diagnosis meeting at which the neuropsychological and clinical data were reviewed. The screening process was standardized with a comprehensive Case Report Form (CRF) recorded for each participant.

Experienced psychiatrists performed all clinical diagnoses. The dementia group consisted of 21 patients with a diagnosis of probable AD or other kinds of dementia based on the Diagnostic and Statistical Manual of Mental Disorders, Fourth Edition (DSM-IV). Diagnosis of MCI was mainly based on the CDR, GDS, and ADL, supplemented by the psychiatrists’ clinical experiences and the scores on the NPI and HIS. The detailed diagnostic criteria included: (1) subjective complaints of memory loss, preferably corroborated by an informant; (2) preservation of general cognitive function; (3) a global CDR score of 0.5; (4) level 2 or level 3 in the GDS; (5) intact activities of daily life (ADL); and (6) an absence of dementia. Both amnestic and nonamnestic MCI participants were included. One hundred and fifteen individuals were diagnosed as having MCI and 865 individuals were deemed cognitively normal. It should be noted that the MoCA-BJ and MMSE were not used to diagnose MCI or dementia in the present study.

### Statistical analysis

Group differences in demographic variables, MoCA-BJ scores and MMSE scores were examined using one-way analysis of variance (ANOVA) or Chi-square analysis. Pairwise comparisons were further performed when necessary, with the significant level adjusted by the Bonferroni method. Cronbach’s alpha was computed in order to measure the internal consistencies of the MoCA-BJ and its sub-domains, and Pearson correlation coefficients between the MoCA-BJ and the MMSE were calculated to index criterion-related validity. We assessed the sensitivity and specificity of the two scales by using the recommended cut-off score of 26 for both the MoCA-BJ and the MMSE. We also reported the cut-off score for optimal sensitivity and specificity based on our findings. Receiver operating characteristic (ROC) analysis was used to assess the effectiveness of the seven cognitive domains involved in the MoCA-BJ in differentiating MCI from normal controls (NC), and dementia (Dem) from MCI. Item analysis was also conducted to assess whether each item was suitable for MCI detection in this population. Regional differences on the MoCA-BJ and the MMSE were analyzed with ANOVAs, and a linear regression was conducted to control for the influence of demographic characteristics on the MoCA-BJ scores. In addition, as statistically significant differences in age and education were found among the three diagnosed groups and the three regional groups, non-parametric analysis of covariance (rank ANCOVA) was then used to compare MoCA-BJ and MMSE scores among the diagnosed groups (NC/MCI/Dem) and the different regional groups (new town/old town/rural area) adjusting for age and education. Area under the curve (AUC) was used to compare the diagnostic performance between the MoCA-BJ and the MMSE. All statistical analyses were conducted using SPSS 15.0 (IBM Corporation, Somers, NY).

## Results

### Demographic characteristics and group differences

Sample characteristic descriptions are summarized in Table 1. Group differences were found in age (*F* (2, 995) = 9.74, *p* < .001) and education (*F* (2, 993) = 21.72, *p* < .001), whereas the gender distribution (*χ*^*2*^ = 2.64, *df* = 2, *p* = .267) was the same across the three groups (i.e., MCI, NC, and Dem). The MCI and the NC were matched in age (*p* = .417), but the MCI had lower education level than the NC (*p* < .001). Dem was the oldest and had lowest education level among all the three groups (*ps* < .010). Group differences were found on both MoCA-BJ (*F* (2, 998) = 97.85, *p* < .001) and MMSE (*F* (2, 998) = 134.90, *p* < .001). These group differences were further analyzed with age and education as covariates and group differences on the MoCA-BJ and the MMSE remained significant (Table 1). Post-hoc analysis indicate that both scores on the two brief screening tools showed significant group differences (*ps* < .001), with a highest score for the NC and a lowest score for the Dem.

**Table 1:** Demographic information and mean scores of MoCA-BJ and MMSE

	**NC (n = 865)**	**MCI (n = 115)**	**Dem (n = 21)**	***p-*value**	**Adjusted *p*-value**^**c**^
Age	70.40 ± 7.13	71.45 ± 7.26	77.10 ± 8.01^a^	< .001	
Education	10.48 ± 5.33	8.43 ± 5.46	3.80 ± 4.75^b^	< .001	
% Female	56.5%	59.1%	71.4%	.267	
MoCA-BJ	22.29 ± 5.36	17.78 ± 6.29	8.10 ± 6.36^b^	< .001	
Adjusted MoCA-BJ, mean ± SE^c^	22.37 ± .22	17.78 ± .61	8.12 ± 1.66		<.001
MMSE	26.58 ± 3.82	23.30 ± 5.4	13.19 ± 6.74^b^	< .001	
Adjusted MMSE, mean ± SE^c^	26.64 ± .16	23.30 ± .60	13.35 ± 1.84		<.001

*Notes:* NC = Normal Controls; MCI = Mild Cognitive Impairment; Dem = Dementia; MoCA-BJ = Beijing Version of the Montreal Cognitive Assessment (scores include education correction); MMSE = Mini-mental State Examination.

^a^ D > NC = MCI; ^b^ D < MCI < NC; ^c^*p*-value obtained from non-parametric analysis of covariance with adjustment for age and education.

### Psychometric properties and item analysis of the MoCA-BJ

Cronbach’s alpha of the MoCA-BJ was 0.88, suggesting a good internal consistency. There was a high correlation between MoCA-BJ and MMSE (*r* = 0.83, *p* < .001), indicating a good criterion-related validity. Further analysis found that the internal consistencies of sub-domains were relatively low in naming and sentence repetition tasks (naming: Cronbach’s α = 0.55; sentence repetition: Cronbach’s α = 0.41), with the lowest scores on the naming item “Rhinoceros” and the repetition item “I only know that Liang Zhang is the one to help today” compared with other items in each task.

Item analysis revealed that except the last item “City” in the orientation domain showed high accuracy in both the MCI and the NC groups (*χ*^*2*^ = .69, *df* = 1, *p* = .408), all the other items were successful in discriminating the two groups. With regard to the discrimination between the MCI and Dem, the following items with low scores in both two groups failed to yield significant group differences: trail making (*χ*^*2*^ = 1.32, *df* = 1, *p* = .251), one item in the sentence repetition task (i.e., “I only know that Liang Zhang is the one to help today”; *χ*^*2*^ = 2.91, *df* = 1, *p* = .088), and two items in the delayed recall (i.e., “velvet” , *χ*^*2*^ = 2.03, *df* = 1, *p* = .155; and “daisy”, *χ*^*2*^ = 2.16, *df* = 1, *p* = .142).

**Figure 1:**
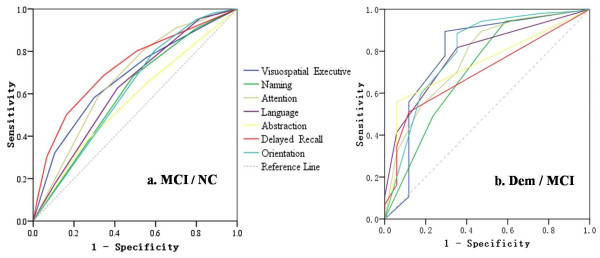
ROC curves for seven cognitive domains of MoCA-BJ to detect: a. MCI from NC; b. Dem from MCI.

ROC curves (Figure 1) were drawn to determine the discriminatory validity of the seven cognitive domains of MoCA-BJ for MCI versus NC, as well as MCI versus Dem. Areas under the curve (AUC) of delayed recall (0.72, 95% CI: 0.67 - 0.77) was the largest for the discrimination between MCI and NC (Figure 1a). With regard to the discrimination between MCI and Dem, the orientation (0.80, 95% CI: 0.67 - 0.93) and visuospatial/executive (0.79, 95% CI: 0.65 - 0.94) domains demonstrated largest AUCs in comparison to the other cognitive domains (Figure 1b).

### Sensitivity and specificity of the MoCA-BJ for MCI detection and its comparison with the MMSE

**Figure 2:**
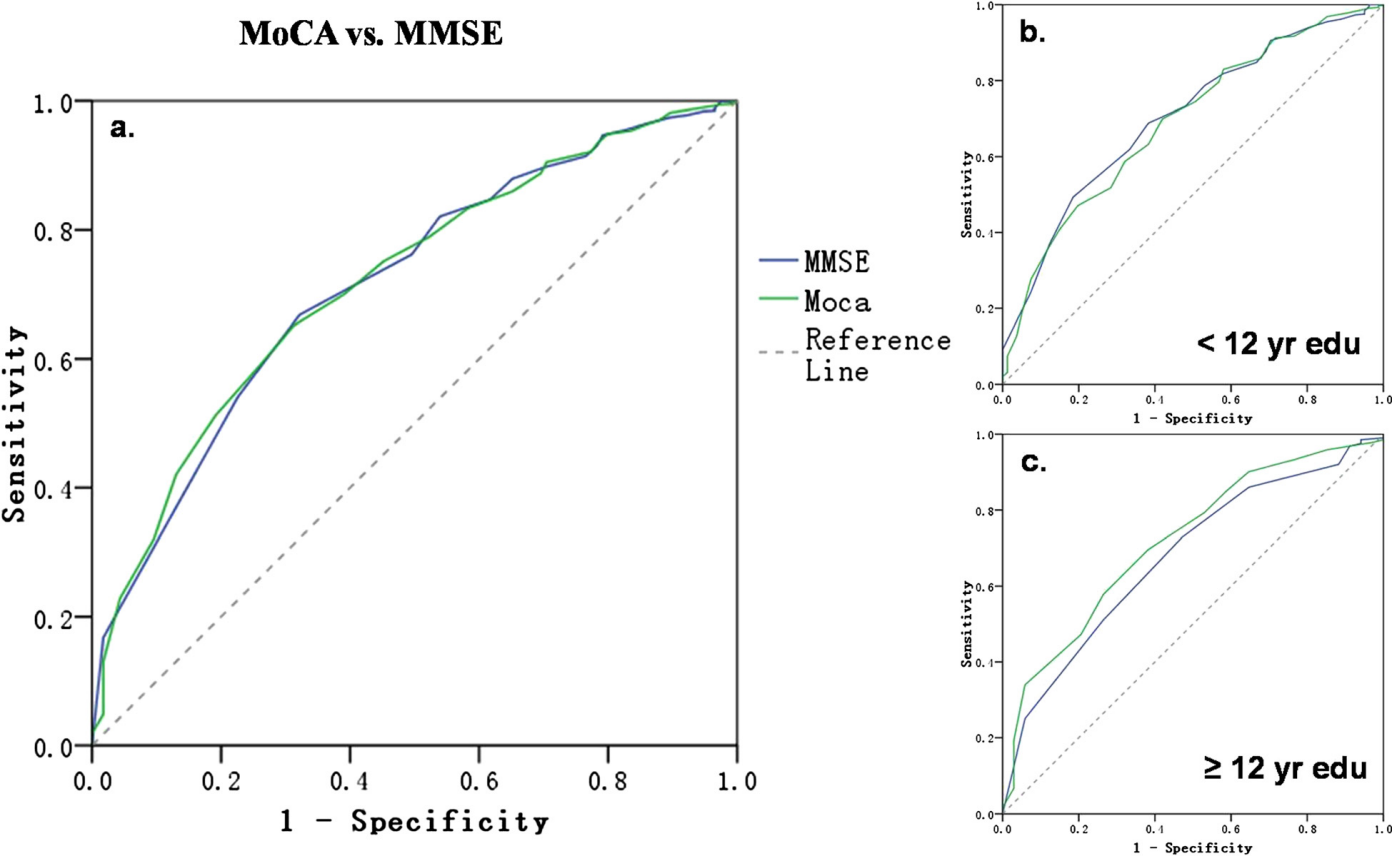
ROC curves for MoCA-BJ and MMSE to detect MCI from NC and Dem in: a. all the sample; b. a sub-sample of low education level (i.e. education years < 12 years); c. a sub-sample of high education level (i.e. education years ≥ 12 years).

The ROC analysis of the MoCA and the MMSE revealed similar AUCs (MoCA-BJ, 0.71, 95% CI: 0.66 - 0.75; MMSE, 0.70, 95% CI: 0.66 - 0.75), suggesting the equivalent diagnostic accuracy of the two scales in detecting MCI (Figure 2a). However, the ROC analysis of the MoCA showed improved diagnostic accuracy in the sub-sample with higher education, despite that the MoCA-BJ scores been already been corrected for education (i.e. adding one point if ≤12 year education). For the participants with less than 12 years of education (*n* = 546), the AUCs of MoCA-BJ and MMSE were 0.67 and 0.68 respectively, both of which were lower than those in the entire sample. In contrast, for the sub-sample with higher education (*n* = 451), the AUC of MoCA-BJ was larger (0.72) than that of MMSE (0.68), suggesting a higher diagnostic accuracy for the MoCA-BJ (see Figure 2c).

Using the recommended cut-off score of 26, the MoCA-BJ showed a high sensitivity of 90.4% but a low specificity of 31.3%, whereas the MMSE showed a relatively lower sensitivity of 50.4% but a higher specificity of 74.5%. In the present study, the cut-off score for the optimal sensitivity and specificity to detect MCI appeared to be 21/22 for MoCA-BJ, at which the sensitivity and specificity were 68.7% and 63.9% respectively.

### Applicability of the MoCA-BJ in different regions of Beijing

There were significant differences in age (*F* (2, 995) = 32.31, *p* < .001), education (*F* (2, 993) = 189.66, *p* < .001), and the MoCA-BJ (*F* (2, 998) = 97.85, *p* < .001) and MMSE (*F* (2, 995) = 32.31, *p* < .001) scores among the three sub-samples (i.e., New Town, Old Town, and Rural Area of Beijing). However, after adjusting for age and education, the three regions no longer differed in MoCA-BJ and MMSE scores (Table 2). Furthermore, a linear regression analysis indicates that the regional effect disappeared (*β* = −.03, *t* = −.95, *p* = .340) after controlling for age and education. This suggests that the regional differences in demographic characteristics could account for some variances on the MoCA-BJ.

**Table 2:** Area differences in demographic information, prevalence of MCI and dementia, and mean score of MoCA-BJ and MMSE

	**New Town (n = 573)**	**Old Town (n = 238)**	**Rural Area (n = 190)**	***p-*value**	**Adjusted *p*-value**^**c**^
Age	70.24 ± 6.30	73.57 ± 8.12	68.29 ± 7.42^a^	< .001	
Education	12.56 ± 4.84	7.22 ± 4.97	6.30 ± 3.42^b^	< .001	
% Female	53.5%	57.1%	62.6%	.083	
% MCI	10.1%	11.8%	15.3%	.113	
% D	1.4%	3.4%	2.6%	.113	
MoCA	23.24 ± 4.95	18.58 ± 6.83	19.78 ± 5.93 ^b^	< .001	
Adjusted MoCA-BJ, mean ± SE^c^	20.28 ± .47	18.71 ± .45	12.33 ± .62		.086
MMSE	27.02 ± 3.52	24.08 ± 5.78	24.89 ± 5.01^b^	< .001	
Adjusted MMSE, mean ± SE^c^	25.31 ± .42	24.02 ± .40	19.08 ± .55		.624

*Notes:* NT = New Town; OT = Old Town; RA = Rural Area.

^a^ OT > NT > RA; ^b^ NT > OT = RA ; ^c^*p*-value obtained from non-parametric analysis of covariance with adjustment for age and education.

## Discussion

To date, there is a lack of validation studies on the MoCA-BJ scale conducted with a large community-based Chinese sample from both urban and rural regions. The present study provides evidence with population-based sample to demonstrate that the most widely used version of the MoCA in mainland China (i.e., MoCA-BJ) has good internal consistency and criterion-related validity in general, and is fairly reliable to differentiate MCI from normal aging and dementia. The MoCA-BJ as a brief screening tool for fast detection of MCI could be used as a reference by Chinese frontline clinicians.

Consistent with the original MoCA report [6], among all the seven cognitive domains, the delayed recall task was most impaired among individuals with MCI in comparison to NC, and is most sensitive in the differentiation of MCI from NC. On the other hand, the most sensitive domains in discriminating MCI from dementia were the orientation and visuospatial/executive domains. These results indicate that episodic memory seems to decline the most at an early stage of MCI. With the progression of the disease, more basic cognitive functions, such as orientation, visuospatial/executive functioning, show larger declines than memory, since that memory impairment might have reached the “floor” at certain stage of the disease.

Detailed analyses suggest that some items may need further modifications. First, the adapted trail making task in the MoCA-BJ requires switching between Arabic numbers and Chinese sequencing-meaning characters, which is more difficult than the original task that requires switching between numbers and alphabet letters. This is because Chinese sequencing-meaning characters are lower in familiarity in China in comparison to English sequencing-meaning letters (i.e., English alphabets) in English-speaking countries, and thus the performance on this task did not differ between MCI and dementia participants. The trail making task used in the Hong Kong/Changsha version of the MoCA [21,22] requires switching between numbers presented against different background colors. This task is more user-friendly for the illiterate or low-education population, and thus is considered to be more suitable for MCI screening for Chinese older adults. Second, the pictures used in the naming task and the words used in the delayed recall task were directly taken from the original English version without any culture-specific adaptations. “Rhinoceros” in the picture naming task is rarely seen in China and “velvet” and “daisy” in the delayed recall are also unfamiliar to Chinese older adults. Therefore, the naming task showed a low internal consistency and these two items (i.e., velvet & daisy) showed low memory scores. Third, the performance of one item of the sentence repetition task (i.e. “I only know that Liang Zhang is the one to help today”) was so poor that only twenty percent of the population could successfully repeat it, because the literal translation of this sentence from English to Chinese has resulted in an awkward sentence structure in Chinese. There is a need for some adaption to this sentence repetition item.

Among the demographic variables, age showed a negative correlation whereas education level showed a positive correlation with the MoCA-BJ scores. Among the three regions, older adults in the New Town area scored highest on the MoCA-BJ, presumably driven by their significantly higher education levels. In contrast, older adults in the Old Town region had the poorest performance, presumably due to their older age. After adjusting for the demographic confounding variables (i.e., age & education), the correlations between the regions and the MoCA-BJ performances disappeared, suggesting an equivalent applicability of the MoCA-BJ in both urban and rural populations.

The MoCA-BJ has a high sensitivity in the detection of MCI using the recommended cut-off score 26, whereas the specificity is fair, which may increase the probability of false positive diagnoses. This finding is consistent with the results from the two surveys introduced earlier which were conducted in Chengdu [17] and Xuzhou [16] respectively. These studies also indicate a high sensitivity (Chengdu: 98.11%; Xuzhou: 94.70%) and a fair or even low specificity (Chengdu: 26.72%; Xuzhou: 20.10%) of the MoCA-BJ scale when using the recommended 26 cut-off score to detect community-based MCI. In addition, similar to what we found in the current study, researchers in the Chengdu study also recommended a cut-off score of 21/22, even though they did not report the corresponding optimal sensitivity and specificity. However, in contrast to Lu’s study [17], our data did not support the MoCA-BJ as a very valid screening tool for MCI detection in the Chinese population. This discrepancy between the current study and Lu’s study may be due to the following reasons. First, the two studies used slightly different assessment tools. Although Lu et al. claimed that they administered the Beijing version of the MoCA in their study; the items used in their study were actually a combination of the items in the Beijing version and Changsha version of the MoCA. As noted in their description of the assessment, Lu and colleagues changed at least one item- *velvet* in the delayed recall task to *silk* (as in the Changsha version). This modification could improve the diagnostic accuracy due to this culture-specific modification. Second, the two studies involved different samples. The prevalence of MCI was about 20.0% (1687/8411) in Lu et al.’s study but only about 11.5% (115/1001) in the current study. Our sample is a closer match to the pooled MCI prevalence of 12.7% in the Chinese population as reported in a recent meta-analysis [20]. This suggests that our sample could be more representative than Lu et al.’s. Third, the MCI diagnostic criteria in the two studies were different. Their diagnosis was mainly based on the CDR scores, whereas ours was made upon a more comprehensive and relatively stricter criteria, under which a diagnosis of MCI was determined not only based on the CDR scores, but also on the GDS scores, and supplemented by the NPI and HIS scores, and more importantly, on the psychiatrists’ clinical experiences. These differences in diagnostic criteria might also have contributed to the differences in the prevalence of MCI in the two studies. In summary, all these differences mentioned above could lead to the inconsistent conclusions between Lu et al.’s study and ours.

It should also be noted that the MoCA was not demonstrated to be superior to the MMSE in current study as other studies did [6-9,23] in MCI identification, while the MoCA-BJ was indeed found to be a slightly more sensitive screening tool than the MMSE in a sub-sample with higher education (Figure 2). The relatively low education level of Chinese older adults (mean education years = 10.05 ± 5.45) and the items with cultural and linguistic differences mentioned above could have contributed to these inconsistent findings. Due to the cultural and language differences and the low education level of Chinese elderly, some items that are reliably administered in Western countries to differentiate individuals from different diagnostic groups may not apply to the Chinese population [24].

This study has several limitations. First, the given sample sizes of NC, MCI and dementia groups were different. Moreover, the number of participants in the dementia group was very small, which might have compromised the statistical ability of our between-group comparisons [19]. In a similar vein, the small sample size in the rural area might also have weakened the conclusion drawn from this sample. In addition, the study was only conducted in Beijing. China is a large country with considerable regional variability in several sociocultural domains, such as economy, custom, climate, and diet, which could influence the performance of local residents on the neuropsychological tests. We recommend future studies to recruit participants from more diverse regions of the country.

## Conclusions

The MoCA-BJ, in general, is an acceptable tool for MCI screening in both urban and rural regions of Beijing. However, presumably due to the linguistic and cultural differences between the original English version and the Chinese version of the scale, and the lower education level of Chinese older adults, the MoCA-BJ was not found to be better than the MMSE in MCI screening. Further modifications to a few items of the MoCA-BJ are necessary for the scale to be more suitable for the Chinese population, which could then improve the effectiveness of the MoCA-BJ in MCI screening in China.

## Competing interests

The authors declare that they have no competing interests.

## Authors’ contributions

JY and JL conceived the study design. JY participated in the data collection, performed the statistical analysis, and drafted the manuscript. JL is the principal investigator of this project, and supervised the statistical analysis and the manuscript writing and revision. XH is the coordinator of this project and contributed at all stages of the data collection. All authors read and approved the final manuscript.

## Pre-publication history

The pre-publication history for this paper can be accessed here:

http://www.biomedcentral.com/1471-244X/12/156/prepub
